# Peer-Led Models Focussed on Emotional Distress and Suicide Prevention: A Scoping Review

**DOI:** 10.3390/ijerph23020273

**Published:** 2026-02-23

**Authors:** Dianna G. Smith, Mel Giugni, Amelia Gulliver, Scott J. Fitzpatrick, Heather Lamb, Louise A. Ellis, Erin Oldman, Helen T. Oni, Caroline Allen, Michelle Banfield

**Affiliations:** 1 Centre for Mental Health Research, National Centre for Epidemiology and Population Health, The Australian National University, Canberra, ACT 2601, Australia; m.giugni@latrobe.edu.au (M.G.); amelia.gulliver@anu.edu.au (A.G.); scott.fitzpatrick@anu.edu.au (S.J.F.); michelle.banfield@anu.edu.au (M.B.); 2 Olga Tennison Autism Research Centre, School of Psychology and Public Health, La Trobe University, Bundoora, VIC 3086, Australia; 3 Centre for Healthcare Resilience and Implementation Science, Australian Institute of Health Innovation, Macquarie University, Sydney, NSW 2109, Australia; louise.ellis@mq.edu.au; 4 Lifespan Health and Wellbeing Research Centre, School of Psychological Sciences, Macquarie University, Sydney, NSW 2109, Australia; 5 Roses in the Ocean, Newstead, QLD 4006, Australia; erin@rosesintheocean.com.au; 6 Sonder, Elizabeth, SA 5112, Australia; 7 Department of Public Health, School of Psychology and Public Health, La Trobe University, Bundoora, VIC 3086, Australia; 8 Independent Lived Experience Consultant, Sydney, NSW 2000, Australia; 9 The ALIVE National Centre for Mental Health Research Translation, The Australian National University, Canberra, ACT 2601, Australia

**Keywords:** peer work, suicide, suicide prevention, crisis, distress, models of care, scoping review

## Abstract

**Highlights:**

**Public health relevance—How does this work relate to a public health issue?**

- Suicidality represents a significant and persistent public health concern
- Peer-led models/interventions are a key and growing component in suicide prevention.

**Public health significance—Why is this work of significance to public health?**

- Training and supervision are essential and should be based on peer work principles.
- Insufficient reporting of peer roles and lived experience involvement.

**Public health implications—What are the key implications or messages for practitioners, policy makers and/or researchers in public health?**

- More extensive research on peer-led models is needed to build a robust evidence base.

**Abstract:**

Suicidality is a significant and persistent public health concern, and people who are suicidal report negative experiences with clinical services. Peer-based interventions are a rapidly growing component of mental health care and suicide prevention. This scoping review’s aim is to identify, summarise and synthesise the design, features and evidence for peer-led models and interventions for people experiencing emotional distress or suicidal crisis. This study followed the Joanna Briggs Institute scoping review guidelines. Online databases were searched in May 2022 and in October 2024. A total of 59 papers were identified. The scoping review provides an overview of key components of service models and interventions. In general, peer-led programs were widely accepted, with participants reporting positive improvements to mood, social connectedness, communication and coping skills. Despite the importance of training and supervision, a review of training content revealed a discordance between training and peer work principles in some cases. A concentration on facilitation of the service model or intervention rather than on the peer model itself meant there was limited information on the empirical and ethical arguments that supported the model of care. Future research is needed on peer-led models and how involvement and engagement of peers, consumers and carers can positively influence the planning, design, implementation and evaluation of new service models and interventions.

## 1. Introduction

Suicidality represents a significant and persistent public health concern. In Australia, the lifetime prevalence of suicidality in people aged 16–85 years has been estimated at 16.7% for suicidal ideation, 7.4% for a suicide plan, and 4.9% for a suicide attempt [1]. Many factors contribute to the lack of adequate support for people experiencing suicidal crisis or distress. Structural and environmental barriers, such as limited availability of crisis care and support, safety and privacy concerns and long wait times, have all been identified as reasons for poor service user experiences and outcomes [2,3,4]. Research has also shown that people who self-harm report negative experiences with clinical services, including discriminatory and culturally inappropriate forms of care and unmet psychosocial support needs [5,6]. These poor experiences not only discourage future help-seeking but may also exacerbate self-harm risk [5,7]. 

Clinical environments such as Emergency Departments (EDs) are ill-suited for people experiencing acute distress. Long wait times, increased noise levels and the sterile hospital environment have been shown to increase a person’s distress [8]. In addition, EDs are frequently perceived as providing perfunctory care only and are unable to meet the needs of those seeking help for suicidality or self-harm [2]. Alternatively, non-clinical environments have been shown to be more responsive to the needs of service users and preferred sources of support for those in distress [9,10]. Non-clinical environments can promote recovery and wellbeing by reducing stress and anxiety, boosting social connectedness and strengthening people’s sense of belonging, safety and personal meaning [11,12]. Non-clinical settings may include informal health care settings, drop-in centres and ‘Men’s Sheds’, but also extend to include ordinary, everyday ‘therapeutic spaces’ such as gardens, libraries and churches [11]. 

The emergence of non-clinical approaches to suicide prevention coincides with the international expansion of peer models within community mental health initiatives [13,14]. Peers are a distinct type of support professional who use their own lived experience to inform their practice and support others in their recovery. Peer work is differentiated from other support work by its recovery-oriented perspective that purposively uses positive self-disclosure and role-modelling to inspire hope and develop an equal and reciprocal relationship [15]. Underscored by peer work principles, peer models include a diverse range of service delivery approaches ranging from mutual support groups through to individual programs provided by trained peer workers [16]. Peer work may serve different functions, such as aiming to reduce stigma, providing crisis support and respite, and recovery-oriented support, including relationship building and connection [13,16,17]. Peer-based approaches are an increasingly prominent element of suicide-prevention strategies in health services. They create care pathways for people who may not seek conventional mental health support or who are ineligible for acute crisis services, an issue that is growing across Australian mental health systems [18,19]. 

‘Safe spaces’ or ‘safe havens’ as they are also known, are peer-led alternatives co-designed with people with lived experience of distress that offer safe, accessible, recovery-oriented support to people in distress with or without suicidality [20]. The safe haven model was first implemented in the UK and has since been trialled in Australia [21]. The key features that underpin these models of care are broad and multifaceted; however, little is known about their scope, design and the evidence for peer-led models specifically focused on distress with or without suicidal crisis.

Interest in peer-based mental health approaches has resulted in multiple systematic reviews over the last decade [22]. These reviews have examined peer-based approaches for suicide prevention [16,19], for people with serious mental illness [23,24,25] and for specific populations, e.g., youth, perinatal women [26,27]. However, to the authors’ knowledge, no reviews have focused broadly on distress, either with or without suicidal crisis, nor specifically on the characterisation of the ‘peer model’ itself, i.e., those that are led, informed, co-designed or co-facilitated by peers with lived experience of emotional distress and/or suicidal crisis. In addition, this review provides a more comprehensive scope by incorporating interventions that focus on emotional distress or crisis in addition to suicidality. The aim of this review, therefore, is to identify and clarify the range of features of peer-led models for those experiencing emotional distress with or without suicidal crisis, their design, who was involved in the design and conduct of the research and the evidence to support these models.

## 2. Materials and Methods

### 2.1. Rationale

Scoping reviews are recommended for identifying and analysing certain characteristics or concepts that underpin a field of research or topic [28]. They are useful for exploring research activity in areas that encompass multiple sources of evidence and research methodologies [29]. The methodological framework of Peters et al. [28] was used to guide the scoping review. 

This nine-stage approach includes the following stages:

1. Defining and aligning the objective/s and question/s
2. Developing and aligning the inclusion criteria with the objective/s and question/s
3. Describing the planned approach to evidence searching, selection, data extraction, and presentation of the evidence.
4. Searching for the evidence.
5. Selecting the evidence.
6. Extracting the evidence.
7. Analysis of the evidence.
8. Presentation of the results.
9. Summarising the evidence in relation to the purpose of the review, making conclusions and noting any implications of the findings.

The PRISMA-ScR (extension for Scoping Reviews) reporting checklist [28] is presented in Supplementary Information: Table S1.

### 2.2. Scoping Review Questions

As a scoping review, our primary research question was intentionally broad to capture a wide range of data:

1. What are peer models of support for people experiencing emotional distress or suicidal crisis?

Secondary questions were as follows:

2. How have peer, consumer, and carer inputs and co-design processes informed the development of peer models?
3. What is the evidence to support these models?

This final question included examining data on model implementation as well as effectiveness.

### 2.3. Study Selection

#### Inclusion and Exclusion Criteria

Table 1 lists the criteria upon which sources were considered for inclusion in the scoping review, which was developed using the PCC Framework (Participants, Concept, Context) [28]. Papers that explicitly investigated, reported or discussed any aspect of the implementation of peer-influenced models for managing and supporting people experiencing emotional distress and/or suicidal crisis, as indicated in the title, keywords or abstract, were included. Searches were limited to English-language peer-reviewed articles, with no restriction on publication date or study design (quantitative, qualitative or mixed-methods). No grey literature was included in this review due to resourcing and time constraints. A review of grey literature in this area can be found in a recent systematic review on crisis support services as alternatives to emergency departments [30].

### 2.4. Search Strategy

The search strategy was iteratively developed by the authors with the assistance of an experienced academic librarian. Search terms were based on a combination of established search terms from previous search strategies and Medical Subject Headings (MeSH) for suicide AND crisis intervention AND peers. Boolean operators and proximity operators were used to maximise the sensitivity and precision of terms searched, and “wildcards” including truncation and nesting were employed to account for variations, plurals and spelling. The following electronic databases: PsycINFO, MEDLINE (both via the OVID interface), CINAHL, Web of Science and Scopus were originally searched in May 2022. Due to the length of time elapsing in reviewing all the articles, an updated search was conducted in October 2024 to ensure the review included current research. Supplementary Information Table S2: Key search terms and search strategy presents the search terms and the complete search strategy for MEDLINE.

The search strategy was designed to capture a comprehensive selection of studies describing both peer models of care for people experiencing emotional distress without explicit mention of suicidal ideation and crisis (hereafter referred to as non-suicide-specific) and peer models of support for people experiencing suicidal crisis, ideation and distress (hereafter referred to as suicide related). This broad scope allowed the researchers to examine similarities and differences between the areas to inform the implementation of models such as safe spaces and safe havens.

### 2.5. Screening and Identification of Studies

**Figure 1:**
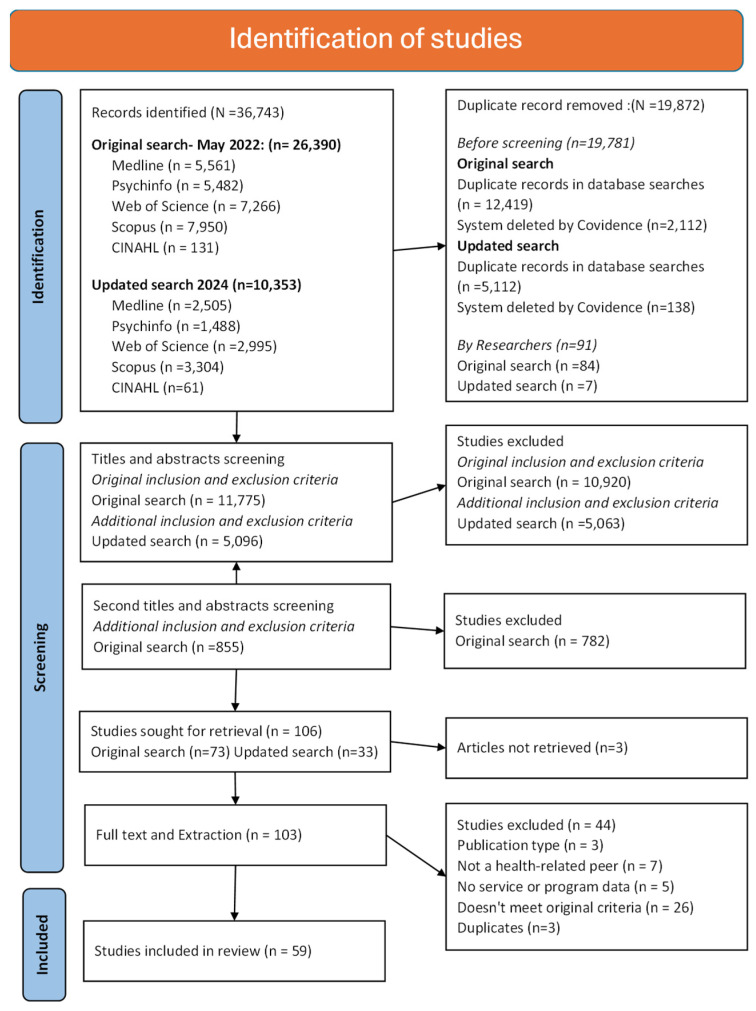
PRISMA flowchart of the study selection process.

Figure 1 presents the PRISMA flow diagram. This review was undertaken by two teams: a pilot team (MG, SJF, HL) and a review team (DGS, ES, CC, AG, EO, HTO, ARM, JC, GS, CA, ZF, LE, AF, CR, GR, MB). All identified records were downloaded to EndNote 20 Reference Manager (Clarivate Analytics, PA, USA) and imported into Covidence systematic review software (Veritas Health Innovation, Melbourne, Australia). Duplicate citations were removed using Covidence. All screening and extraction were performed using Covidence. 

One of the co-lead researchers (MG) conducted the first database search (26,390 records), and the other co-lead researcher (DGS) conducted the updated search (10,353 records); duplicates were deleted in both instances (19,872 records). The eligibility criteria (Table 1) were tested on a random sample of 100 abstracts in the original search until a 75% inter-rater agreement was reached between the three pilot team members (MG, SJF, HL) [28]. 

For the initial search (n = 11,775), all titles and abstracts were screened by two independent reviewers: a member of the pilot team (MG, SJF, HL) and a member of the second review team (ES, CC, AG, EO, HTO, ARM, JC, GS). When consensus was not reached, another review team member was consulted to resolve disagreements. This resulted in 855 records selected to go through to full-text screening. The team discussed the feasibility of synthesising this volume and chose to apply tighter definitions of criteria to reduce the scope. A second screening of titles and abstracts was performed independently by four reviewers (authors, MG, AG, SJF, MB) to narrow the focus to articles that were explicitly about distress and where peers were explicitly health or disability related, as well as to remove records which were not peer reviewed. Table 1 presents the exclusion criteria used at this second screening stage. 

The updated search was completed in October 2024. A greater than anticipated number of articles (n = 5096 after duplicates removed) were found; thus, a modified review of the titles and abstracts was performed for these articles. To perform the modified review, two of the researchers screened the titles and abstract (DGS screened all articles, AG screened a random 10% sample using the full criteria presented in Table 1). Inter-rater agreement was calculated, showing >90% agreement. Those articles that were not agreed upon were verbally discussed and resolved via mutual agreement.

### 2.6. Data Extraction

Full text review and extraction were performed simultaneously by one of the following: MG, SJF, HL, HTO, CA, CC, EO, ZF, LE, AF, CR or GR. They were then independently checked by DGS. Any conflicts were resolved by AG through cross-checking extracted data against the original papers. Forty-four articles were excluded at this stage (Figure 1). 

A data extraction tool was developed in Covidence and refined following coding of a small number of papers. Data were extracted on the following characteristics:

- Article identifiers: title, authors, year of publication, country of origin, study design.
- Type or scope of model, setting, specific target population.
- Details of how the peer model was designed and implemented, and/or delivered, training and supervision, types of people involved, such as those with lived experience, carers and health professionals.
- Details of peers, consumers, caregivers or co-design processes involved at any point of the design of the program, conduct or interpretation of the study
- Outcomes as determined by study measures.

Extracted data were tabulated and analysed to provide a descriptive summary of the types and features of peer-influenced models. 

## 3. Results

A total of 59 articles were included in the review. Findings comprise a summary of data extracted from the studies. Table 2 presents detailed information on the aims of the studies and peer model characteristics. Supplementary Table S3: Characteristics of emotional distress without explicit mention of suicidal ideation and crisis articles (n = 22) and Table S4: Characteristics of peer models of support for people experiencing suicidal crisis, ideation and distress articles (n = 37) provide additional information including country of origin, study design, setting of the intervention, study participants, measures and outcomes, including psychological outcome measures and outcomes on mental health services.

Study characteristics, as detailed below, non-suicide-specific and suicide related articles provided a similar range of information on the study characteristics and peers, and equally limited details on training and supervision. However, suicide related articles provided greater depth on the components of the peer model and the outcomes measured.

The volume of information provided on the characteristics of the peer worker, training and components of the peer models varied in both groups of articles, with one of the suicide related articles not reporting on any of these aspects [31]. 

### 3.1. How Peers and Co-Design Processes Informed the Models

Less than half (47%) of the papers mentioned how they involved people with lived experience. More suicide related articles [32,33,34,35,36,37,38,39,40] stated that peers were involved in both the design of the intervention and the conduct and interpretation of the research. Papers on non-suicide-specific distress most often reported that peer involvement was only in the design of the intervention [41,42,43,44,45,46,47,48]. The level of detail ranged from the mention of people with lived experience participating in developing a platform for the intervention [49] to a short description of peer involvement in each stage of development of an intervention [38,46] to a full description of peer involvement including information on a debate on the positionality of the peer specialists by a scientific advisory board [33]. 

The following symbols are used in Table 2 to illustrate the different levels of peer involvement in the research.

✓ Peer or co-design involvement in the conduct of research only, 

✓✓ Peer or co-design involvement in intervention or program development, 

✓✓✓ Peer or co-design involvement in both intervention development and the conduct of the research.

**Table 2:** Characteristics of studies (n = 59).

Author, Year	Study Aim/Purpose	Peer Worker Description	Training and Supervision	Peer Model Components	Peer-Led Program Findings	Peers Involved
Studies targeting distress in the context of mental ill-health (non-suicide-specific) (n = 22)						
Acri 2021 [41]	Examine the impact of peer-delivered outreach program upon depression, mental health services engagement for child welfare-involved adult caregivers	Female caregiver, high school-level education, employed at the child welfare agency, experience of child welfare system.	Weekly supervision by a PhD-level social worker and a senior peer advocate at the child welfare organization.	Depression screening, psychoeducation, explaining treatment options, support initial contact with referral source.	NR	✓✓
Byrom 2018 [42]	Assess the acceptability, impact of peer support intervention program for university student.	Students, experience of depression not required but was encouraged	A 2-day course (active listening skills, motivational interviewing, boundaries and safeguarding). Regular telephone supervision with Student Minds.	Publicise the sessions, recruit participants, intervention facilitation,	Program was generally well-received, with improvements in confidence in talking about mental health; ability to look after their own mental health and mental health knowledge. Students with lower mental wellbeing at the start of the course were more likely to return for subsequent sessions.	✓✓
Conner 2018 [50]	Evaluate the relationship between peer support and hospital readmissions for recently discharged older adults for a medical condition with an untreated mental health diagnosis of depression	Adults, 61–91 years, previous treated depressive episode and high school education	20 h training (peer support, aging, recovery, communication skills, engaging in mental health treatment, appropriate self-disclosure use, motivational interviewing techniques, crisis management, communicating with healthcare providers), conducted through roleplay, lectures, group discussion and exercises, bi-weekly supervision with project researchers/clinicians.	Motivational interviewing techniques utilisation, shared lived experience, tailored individual support, social support and information, reinforcement of hospital discharge plan, modelling empowerment and control, and write comprehensive contact notes.	NR	NR
Cook 2017 [51]	Summarise the research on peer support benefits for people who have been affected by the suicide of someone in the military, identify evidence- and practice-informed peer support principles and a well-developed program case example.	Suicide loss survivors further along in their grief.	Online workshop, one day face to face training, ongoing in service and specialised training. Supervision by a full-time staff who is a suicide loss survivor.	In the grief camp, Peers partner a child and participate in all their activities. In the 3-day annual seminar they are available for one-to-one sessions with attendees.	NR	NR
Cust 2016 [48]	Identify if support from a peer worker assists in reduction of new mothers’ postnatal depression.	Mothers with mild-moderate postnatal depression. were recovered, not currently receiving psychological support or taking medication.	Apart from training on child protection procedures and confidentiality, no structured training program followed, peers wanted to help from the stance of fellow mum with similar experience. Individual and group support session provided to assist development of support packages	PSW identified the nature of the problem, find a proposed solution, and designed their own proposed ‘support package’	The authors concluded that peers could assist in decreasing new mothers’ postnatal depression. Participants recognised peer workers’ value, peers gave them hope and normality whilst assuring them that they were not failures. Peers mentioned feelings of personal benefit, increased self-awareness and closure. Participants and peers recognised changing perspective, there was no quick fix, it takes time and support	✓✓
Fan 2019 [43]	Examine the feasibility of developing community-based peer service in China for people 18–60 years, diagnosed with schizophrenia or bipolar disorder	Peer workers 18–60 years, with a schizophrenia or bipolar disorder diagnosis, stable for 6+ months; adherent to medications, no drug or alcohol abuse; no severe physical illness, having good social functioning, strong practical skills.	Five half-days training delivered by psychiatrist and clinical psychologist, (peer support services concept and theory, how to design and implement group activities, answer patients’ questions about mental health, handle emergency situations, effective listening and speaking skills). Supervision by doctors; frequency depended on peer worker needs	Two peers facilitated group sessions (daily life skills, social skills, mental disorders, knowledge, entertainment, fine motor skill practice, personal perceptions, healthy lifestyle support, and emotional support).	Participants said that the peer services were beneficial, reported improvements in communications skills and mood, illness knowledge, illness stability. Majority (85%) of peer workers wanted to continue as peer workers, mentioned working skills, communication skills and mood improvements. Caregivers reported confidence in family members recovery, reduction in caretaker burdens and improvement in own mood	✓✓
Gillard 2015 [52]	Develop an empirically and theoretically grounded model on change mechanisms underpinning peer worker interventions for various populations and settings	NR	NR	Shared lived experience, recovery planning, role-modelling recovery, self-care	The authors developed a change model primary mechanism: building trusting relationships based on shared lived experience, peers provided a role model for recovery, facilitated a bridging and engaging mechanism that could lead to strengthening social networks, social functioning, engagement in treatment, hope in the future and empowerment.	✓
Hassouneh 2013 [44]	Test the efficacy of Healing Pathways (HP) program in reducing clinically significantly depressive symptoms in women with physical disabilities.	NR	10-day face-to-face training with the whole team including researchers (crisis intervention, suicide assessment and intervention, understanding depression and anxiety, violence and abuse assessment and intervention, responding to trauma) provided by disability community persons and Psychiatric Mental Health Nurse Practitioners. Other training provided as needed. Weekly group supervision, ad hoc sessions, emergency consults, with Psychiatric Mental Health Nurse Practitioner.	Healing Pathways program sessions facilitation (introduction to the program overview of depression; strengths and goals; mental habits; understanding and managing emotions; sense of self optional content on body image and sexuality, personal identity, role models, violence and abuse, relationships, social support, and physical disability, developing communication skills; stress and coping; wellness; and moving forward)	Participants gave strong, positive feedback of the program’s effect on their lives, maintained that all course content was valuable, suggested the sexuality session be made optional, and that the course was too short to cover all topics, would have liked more personal sharing during the sessions.	✓✓
Jain 2016 [53]	Investigate the perceptions of peer support, support from veterans and support from mental health staff influenced level of engagement in treatment; recovery orientated attitudes toward PTSD and PTSD symptoms.	A VA patient further along in their treatment	No training supplied to Big Brother peers	General mutual support as part of the therapeutic environment, participation in the Big Brother intervention supplying more individualised support to orientate newcomer to program, answer questions, encourage them to participate in and adhere to program.	Whilst Veterans perceived greater support from other veterans than from any other source, their perceptions of support from mental health staff, family and friends, and their Big Brother were mixed. Those reporting negative behaviours by the Big Brother showed less improvement in recovery attitudes. The authors concluded the Big Brother intervention was not a formal peer support model, the Big Brothers were not trained, not better at providing support than any other participant in the program.	NR
Jones 2015 [45]	Evaluate the effects of Home-Start scheme to provide greater understanding of peer-based support schemes for mothers with low mood in 12 months following birth of their child.	Parents with lived experience of depression	40 h training provided	Volunteers use befriending process to deliver the organisation’s social involvement therapy package.	Participants reported on elements that contribute to low mood, social problems, mental health and self-esteem problems, financial issues and parenting challenges. No relation between length of time between initial assessment, engaging with a volunteer, any improvements in the above elements. People left the service because of improvements in wellbeing, peer volunteer no longer working for the organisation, receiving other services.	✓✓
Joo 2016 [54]	Assess the feasibility of peer-delivered depression care for people 50 years and older.	People 50+ years with a history of depression and treatment, 5+ years in recovery.	20 h training (rapport building, communication skills, active listening, experiential knowledge sharing, peer roles, expression of empathy, cultural competence, patient confidentiality, patient safety). Weekly supervision meeting with psychiatrist.	Establish a strong working alliance, identify patient-defined problem, encourage behaviour change, facilitate community and formal mental health services connections.	Participants thought that peers displaying similar experience helped accept credibility of the coping skills. involvement of professionals gave credibility to peer roles, peers gave them hope, practical suggestions, an changes to perspective. Participants whose depression did not improve had perception that intervention was not long enough, had difficulty with trust, and concerns with confidentiality.	NR
Martin 2020 [46]	Assess the feasibility of iHOPE among adults living with cancer.	People affected by cancer in some way.	Maximillian Cancer Support. training	I Hope program facilitation, encouraged participants, stimulated social networking forums discussion, monitored daily social networking posts for safety, reported any technical problems.	Majority (93%) of participants reported the program was easy to navigate, was well managed by the peer facilitators, and the social networking tools were useful.	✓✓
Milne 1989 [55]	Evaluate the pilot study of peer therapists in informal community groups of people experiencing clinical anxiety.	Community-dwelling adults with experience of anxiety, discontinued regular psychologist appointments.	15 h workshop (running a group; counselling; therapy and assessment skills). Weekly supervision meetings becoming less frequent as peers gained in confidence and competence.	Closed group sessions facilitation (anxiety management and rationale; defining and describing anxiety; assessing anxiety; and self-control over anxiety). 10 sessions at least one week apart	The authors concluded that the training course did enhance the peers’ skills and knowledge. The group members were extremely satisfied with how peers ran sessions, progress that they made, and less satisfied with session contents.	NR
Nissling 2020 [56]	Assess patient experiences; feasibility, safety, acceptability; effectiveness of 8-week peer-supported Internet Cognitive Behavioural Therapy (ICBT) program for adults with a diagnosed anxiety disorder	Peer had experience working at an inpatient psychiatric clinic	Training by organisation with an established peer support education program. Weekly supervision from psychologist, discussing the treatment content, the participants’ answers on the questionnaires, and reflections on participants’ written messages.	Provided support, feedback on the therapist-guided ICBT exercises, calling and text messaging the participants.	Participants reported good experiences with peer workers, helped them see another side to anxiety, the possibility of a better future, liked peers checking in with them, and knowing there was someone with similar experiences available to help and understood them. Peers felt satisfied with role, they reinforced the program elements and participants positive behaviours, validating their difficulties, encouraged connection.	NR
O’Connell 2018 [57]	Explore the relationship between peer mentor intervention and improved clinical outcomes, and increased community tenure for adults with diagnosis of schizoaffective disorder, psychotic disorder not other specified, bipolar disorder, major depressive disorder with or without psychotic features	Adults with severe mental illness, self-identified as in recovery, willing to share experiences.	Training (recovery philosophy, promotion, local resources, professional/personal boundaries, safety, cultural competence, gender, trauma-informed care). Supervision provided by study supervisors various methods including weekly team meeting.	Community based support provision.	Recovery mentor group participants reported decreases in drug use, improvements in physical health, hygiene/self-care, unusual behaviour, social functioning and excitement.	NR
Seal 2021 [58]	Determine the effectiveness of telephone motivational coaching to improve veterans’ mental health treatment engagement.	Veterans with mental health issues and prior exposure to counselling.	2-day workshop (motivational interviewing techniques, how to address issues related to suicide prevention, veterans’ mental health recovery, potential suicidal and homicidal ideation, issues related to race/ethnicity, sexual orientation, identity).	Coaching, shared lived experience, motivational interviewing, problem-solving support, resource provision, encouragement, accountability, non-judgemental approach.	Veteran stated benefits included help with problem-solving, suggestions for helpful, practical resources, goals encouragement, that peers were less judgemental, telephones was more convenient than face to face meetings. Between the groups there were similar proportion initiating MH treatment, reported having two+ MH visits. engaging with non-clinician-directed MH treatment.	NR
Suresh 2021 [47]	Investigate peer support as a viable form of support that would benefit university students.	Students who work closely with the university’s mental health services and professionals no mention of lived experience of mental health issues	Training on active listening, open communication, empathy, and crisis management.	Peer workers provide individual support sessions in a safe and supportive environment.	Participants were unlikely to go to other professional services, felt that peers understood them, assisted them with coping skills, realising their own resilience, found the service easy to navigate without many barriers, and would recommend to others. Peer volunteers felt prepared to deal with sessions, and the topics raised. They benefitted from improved self-esteem, and empowerment feelings	✓✓
Tang 2022 [59]	Investigate peer perspective in rendering formal peer support to community-dwelling older adults with depression.	Peer supports aged 50–74, either with depression risk factors or diagnosis.	7-month (100-h) certificate training course (theories and concepts in older adult depression, mental health recovery, peer support communication skills), supplemented with experiential learning and practicum.	Peer support workers involved in telephone engagement, home visits, mental health initial screening, support service users in psychoeducation groups, helped at street booths, community educational events.	Peers shared more about their physical wellbeing than their mental health, established trusting relationship with participants, increased connection, decreased sense of isolation through sharing lived experiences of age and health-related experiences.	NR
Travis 2010 [60]	To evaluate the feasibility of a telephone-based, mutual peer support intervention for people with a current or past diagnosis of depression	Participants had to be in treatment, have a current or past diagnosis of a depressive disorder with ongoing depressive symptoms or disability, have a history of at least two antidepressant trials.	90-min training session on communication skills, self-management practices attended in their pairs. Provided with an intervention manual with conversation guides and suicide intervention and contact details of study staff.	Participants paired together to provide peer support to each other.	Participants reported: satisfaction with the peer support calls; feeling comfortable sharing information; perceiving benefits from the program; expressed appreciation for calling system anonymity; opportunity to speak with someone who understands depression; a chance to provide and receive support from their peers.	NR
Truong 2019 [61]	Understand peer self-disclosure to inform peer training of older adult experiencing depression	Individuals 50+ with history of depression, 5+ years in recovery, previous mental health volunteer experience.	20 h training course by a geriatric psychiatrist, (active listening, relationship building, emotional support provision, encouragement to try something new), used role playing and feedback. Weekly hour-long supervision meetings, peers reported on client progress, received guidance and reinforcement.	8 weeks of depression care	Four primary themes: ‘self-disclosure as a counselling technique’, ‘establishing rapport through personal similarities without direct relation to depression’, ‘showing empathy through experiences of personal struggle’, ‘self-disclosure focused on the peer’. Peer mentors shared personal experiences to guide client action, highlighted similarities of background, experiences, and recent life events, expressed personal understanding of struggles, demonstrated empathy by sharing their experiences of loneliness, grief, health issues, aging. In rare instances, peer mentors shared personal stories focusing on their own distress or experiences. Peer mentors used self-disclosure as a counselling technique (45.1%) to establish rapport through personal similarities; self-disclosure techniques (31.8%): reframing perspectives (17.3%), modelling positive behaviours (14.2%), establishing rapport through personal similarities (32%) and showing empathy (23%).	NR
Vanderkruik 2019 [62]	Explore the perspectives of peer-delivered model to treat depression among Latina mothers.	Participants were asked what peer characteristics they want, bilingual peers, with similar experiences, i.e., older mother with history of depression	Specified that peers needed training, strong supervision and clear system of referral.	There is no explanation on model although peers were asked their opinion of it	Key informants and participants viewed the peer model favourably, flexibility of setting, frequency of contact fitting participants’ preferences was preferred. They emphasised the importance of cultural considerations needed to be incorporated into the design and application of the model. Concerns were noted around confidentiality, supervision, retention of peers and participants.	NR
Wain 2009 [63]	Describe the development of person focused recovery service for people with mental distress.	Experts by experience	NR	12 steps workshops, two steps explored each workshop. Wellbeing workshops to improve mental fitness. Moving forward course assisting people in employment and education.	Authors claimed three evaluations provided evidence of decreases in GP visits, lessening of suicidal feelings and of being an accessible service.	✓✓✓
Studies targeting suicidal crisis or ideation and distress (suicide related) (n = 37)						
Acarturk, 2022 [64]	Assess the feasibility, acceptability, impact, cost of Group Problem Management plus (gPM+) program for Arabic speaking Syrian adult refugees in Türkiye	Arabic speaking refugees (12+ years education) gender matched to group led.	8-day training program. Weekly local group supervision by certified gPM+ trainers.	Intervention facilitation, (manual with case examples, practice skills exercises, problem-solving, stress management, peer-to-peer support)	Participants reported acceptability of strategies provided and the format of the intervention (e.g., group sessions, peer facilitators, gender matching). shared lived experience (participants and facilitators all Arabic speaking with refugee experience).	NR
Alvarez-Jimenez, 2020 [49]	Assess the feasibility, acceptability, safety of Moderated Online Social Therapy plus (MOST+) service for people 16–25 yrs	Trained young people with experience of mental illness	Weekly supervision by research team	General guidance, peer-to-peer support, guide problem-solving discussions, seed discussion threads, encourage participants to define the problem, identify pros and cons and summarise possible solutions, post links to therapeutic tips and resources.	Participants who had full access to the program (including the peer social web) reported more positive experience and higher retention than the group with no peer access.	✓✓
Atif, 2016 [65]	Explore the acceptability of peer volunteers delivering a psychosocial intervention for perinatal depression for pregnant women/mothers, 18–45 years with child <3 months.	Women with 10 years education who shared similar socio-demographic characteristics and life experiences with the target population.	4-day classroom + 2-day field training (counselling skills, perinatal depression, learning behaviour activation and problem-solving techniques). Fortnightly group and field supervision.	Intervention facilitation.	All stakeholders said peer volunteers were acceptable for delivering intervention. Attributes of the peers e.g., being local, empathic, approachable, trustworthy, having similar experiences of motherhood, enjoying a good reputation and motivation contributed to acceptability. Factors influencing peer motivation were effective training/supervision, perception of personal gain, endorsement from their family/community. Barriers included: women’s lack of autonomy, cultural beliefs around perinatal period, stigma of depression, mothers’ lack of engagement and family resistance.	NR
Biggs, 2015 [66]	Explore callers’ experiences of Perinatal Depression Helpline for women experiencing perinatal mental illness and their partners	Volunteers who experienced, or supported someone who experienced perinatal mental illness	24 h training (perinatal mental health, loss+grief, attachment theory, parenthood, values, self-care, counselling skills, Helpline systems, risk-assessment), Applied Suicide Intervention Skills training, Observation of trained peers and counsellors. Volunteer coordinator was present to support volunteers	The peers offered information, support and referral services.	Callers reported positive experiences, feeling better emotionally, the helpline gave information they trusted, emotional, practical and parenting support. Talking to someone with lived experience was main difference between this helpline and others they had accessed, this helped them feel understood and supported.	NR
Bologna, 2011 [67]	Compare mental health clients with acute psychological distress experiences of a peer-run hospital diversion program (PRHDP) with previous experiences in non-peer-run acute inpatient psychiatric program	Professional and paraprofessional consumers who provide case management and crisis intervention services.	NR	Peers collaborate with participants in developing recovery plans, provide mutual support and empathetic listening, crisis and distress tolerance interventions, collaborate with mental health agencies.	The participants stated that peer-run model offered more beneficial services than acute care inpatient services, peers were more available, respectful and supportive, provide companionship, mental health feedback, peers model recovery. The model was more conducive to recovery, reduces mental health stigma, provide private space, allows for personalized schedules. Positive beliefs about peer support were associated with higher levels of social involvement and life satisfaction.	✓
Bonkiewicz, 2018 [68]	Assess the effectiveness of Respond, Empower, Advocate, and Listen (REAL) program for individuals who experienced a police-abated mental health crisis	Person with mental illness who is a trained mental health advocate.	NR	Peers: listen to individuals’ experiences, concerns, and challenges, develop mental health plans, create supportive environment, advocate for them in accessing resources and services, provide referring officers updates on progress, develop ongoing support plans	Being referred to the program was associated with a reduction in future mental health calls for service incidents 24 months after a crisis and associated with a decrease in the odds of being taken into emergency protective custody 12, 24 and 36 months after a crisis.	NR
Brasier, 2022 [32]	Explore the benefits, limitations of peer workers supporting adults experiencing mental distress attending a metropolitan public hospital Emergency Department (ED) with an existing Peer program.	Consumer mental health peer workers, aged 18+ yrs working in an ED	NR	Peers contribute with listening, de-escalation, relationship building skills, using empathy.	Peer workers contribute important skills, their involvement can lead to significant improvements in hope, personal recovery, empowerment, quality of life, reduced hospital admissions, increased satisfaction with support, they could benefit the emergency department staff and organisation by promoting personal recovery, challenging prejudice. Barriers and concerns included: ED culture difficult to change, impact on peers in the long term, Peers face inequitable conditions including pay, discrimination, prejudice. Workforce supports: training; supervision; professional networks; career progression; are important to long-term success.	✓✓✓
Burns-Lynch, 2001 [69]	Evaluate the adoption of peer-based hospital diversion program for people experiencing serious mental health distress but do not necessarily require psychiatric hospitalization	NR	NR	Services provided by staff (peer and mental health specialists): peer-to-peer support, shared lived experiences, clinical monitoring, crisis support, decision-making, resource facilitation.	Participants were satisfied with the service, they would return in the future, the most important services were overnight shelter, meal/beverages counselling, referral. Providers expressed high level of satisfaction with program, professionalism, staff’s ability to help consumers with problems, stated it was a needed service in crisis response system	NR
Chalker, 2024 [33]	Identify barriers, facilitators, perceptions of peer specialists delivering suicide prevention service for veterans with serious mental illness at risk of suicidal crisis, to develop an intervention curriculum	Veteran peer specialists with lived experience of suicide	2 × 4 h training days on intervention content. Bi-weekly group supervision with other veteran peer specialists, research staff, clinical psychologist.	Peers are already engaged in recovery planning with veterans. This intervention included adding safety planning/suicide prevention care (including 4 reminders for living) to their skill set,	Peers mentioned that following the intervention review, they felt more comfortable, confident, competent in undertaking suicide prevention care.	✓✓✓
Cubellis, 2018 [70]	Explore peer workers’ vulnerability that is embedded in experience-informed care at a peer-led psychiatric crisis respite centre	Peer specialists were individuals with lived experience of the psychiatric system	Intentional Peer Support (IPS) training	Peers shared life experiences.	Peers acknowledged that the work that they are doing is necessary, unique, important and were committed to helping others by using their experience. However, the professionalisation and commodification of peer work and the subsequent need to work within a broken system can lead to devaluing of a peer service. It can lead to peers leaving the work that they are committed to because they ca not do the work they want within the constraints of the system.	NR
Dos Santos, 2015 [71]	Investigate the experiences of adult voice-hearers who attend the Hearing Voices Network New South Wales peer support groups.	A trained facilitator, no information on whether the facilitator has lived experience.	Facilitator Training Workshop	Influence group running, assist in setting tone, reinforce guidelines of confidentiality and equality amongst members, utilising resources such as recovery stories and information booklets.	Participants reported no changes in frequency of the voices; however the way they interacted with voices changed, there were improvements in sense of self, learning to live a meaningful life with the voices, increased willingness to talk to others about their experiences. Social connections, value of sharing, desire for more group members were important.	NR
Drouin, 2023 [72]	Provide findings to address multimorbidity of intimate partner violence, suicidality and depression using peer support program for people discharged from ED or inpatient facility or people obtaining services through domestic violence service	Clients who reported a suicide attempt, suicidal ideation, and/or IPV-related events	Peers trained, supervised by a nurse, together they worked as part of a clinical team.	Provide: acute crisis response with caring, rapid follow-up contact; patient safety care transition protocols; a text messaging and phone just-in-time support; support to navigate aspects of recovery, connection to community recovery agencies, treatment and referral to other services.	It was felt the program’s aims of engaging, referring and caring were met with 100% of participants having a safety plan in their records, referrals to mental health care and other community support agencies.	NR
Eikmeier, 2019 [73]	Report on the development of Recovery Café—a peer run service.	NR	The peers were coached by ‘Experienced Involvement’ trainer.	Intervention facilitation	The service provided an important role in the reduction of loneliness, boredom and emotional crisis, participants were found to regularly visit the service, attendance led to social activities outside of Café.	NR
Flegg, 2015 [34]	Evaluate peer-to-peer best practices from the viewpoint of people who had engaged with community-led peer-to-peer services	Members of 3 community-led peer-to-peer mental health services.	NR	NR	Participants felt peer services were more beneficial than other services, enabled knowledge sharing and supportive friendships. Authors concluded whilst peer services potentially provide benefits reducing stigma, they might not be appropriate for those in crisis and should be combined with other services.	✓✓✓
Fletcher, 2020 [35]	Examine experiences of peer staff, non-peer program directors working at the first peer respite in California.	People with lived experience of mental health issues.	Intentional Peer Support training Peers practiced co-supervision.	The peer house manager facilitated discussions on management of day-to-day tasks, job safety, guest safety, and appropriate peers’ roles. Peer staff provided support, planned outings, coordinated meal preparation, linked guests to services	Participants felt they achieved their goals of reducing acute psychiatric crises emergency hospitalizations and service costs, fostering recovery, increasing consumers’ meaningful recovery choices goals. They did not feel they established a true peer-operated and staffed crisis residential program due to systemic constraints that limited program’s autonomy to uphold peer values. Reasons included: having to admit clients not a good fit with the recovery philosophy; recovery paradigms ideological differences; staff evaluation challenges, inconsistent management with arbitrary, non-collaborative decision making. Peers felt they were assimilated into mental health services processes which diminished peer service innovative traits.	✓✓✓
Griffiths, 2019 [74]	Explore women’s experiences of using Listener Scheme to help them manage their self-harm in one women’s prison.	Women prisoners involved in prison therapeutic community	Samaritan training to provide a confidential listening service.	Intervention facilitation	Participants showed preference for professional support over peer support; used already established staff relationships, preferred speaking to staff due to confidentiality concerns. Staff and peers felt both types of support worked hand in hand especially when staff had limited availability.	NR
Heyland, 2021 [75]	Describe the implementation of peer support specialists for people presenting to emergency departments with a mental health issue/crisis	People with lived experience of mental ill-health	NR	Provide support, comfort, encouragement, education, inform patients of alternative services, conduct follow-up phone calls 7, 30 and 60 days after initial visit.	Participants said peers were empathetic, informative, talking to a peer gave them feelings of hope. Having peers in ED was beneficial, two-thirds (64%) said that they would not use the alternative service; 18% returned to ED, two thirds due to suicidal ideation or self-harm and a third for medication.	NR
Johnson, 2018 [76]	Assess if self-management intervention for people leaving the care of mental health crisis teams, reduced subsequent rates of acute care re-admission.	Peers with personal experience of using mental health services	Training (to become familiar with program workbook, listening skills, cultural awareness, self-disclosure, confidentiality). Fortnightly group supervision by NHS clinicians. Also support from the research team.	Assist participants with recovery workbook, setting recovery goals, identifying early warning signs, strategies to maintain wellbeing, establish community functioning and networks to avoid relapse.	NR	✓✓
Klim, 2022 [36]	Gain an understanding of how peer specialists telling recovery stories related to suicide may inform training programs, address concerns about services safety and effectiveness.	People with lived experience of suicidal ideation, at least one year peer specialist experience, state certified	Training program (disclosing story in safe manner, avoidance of excessive details and focus of recovery over illness).	Provide emotional support focused on self-determination, wellness, addressing hopelessness; serve in case management roles to support engagement; navigate services or community resources; lead skill-development groups.	Authors found limited discussions related to suicide. Peers convey lived experiences related to suicide while maintaining a message of recovery. Improve suicide-protective factors such as belonging and hope by sharing experiences of crisis and recovery.	✓✓✓
Kumar, 2019 [77]	Assess the value of services for veterans with posttraumatic stress disorder (PTSD) attending the Veterans Affairs peer support groups	Certified peer specialist veterans, diagnosed with PTSD	NR	Share lived experiences, provide emotional and informational support, facilitate peer groups, peer-led discussions, peer-led recovery planning.	Participants reported positive experiences including flexibility in group sessions, shared experiences contributing to a sense of equality and respect, comfort, trust and camaraderie; improved communication among participants leading to honest sharing and mutual support; became more aware of their struggles and accepted the need to address them; coping tools were seen as helpful in managing emotions and symptoms; connectivity extended beyond the group setting with participants reconnecting with friends and family, feeling more open, engaged in social interactions. Peer service seen as separate entity from other VA mental health resources.	NR
Lawn, 2008 [37]	Present a formal evaluation of South Australian Mental Health Unit’s Peer Service for adult mental health services users, either discharged from hospitals or at risk of hospitalization due to mental health conditions.	Individuals with lived experience of mental health conditions, hospital admissions experience, managing their own recovery effectively, mental health system understanding.	Certificate course in community services; 6-week peer worker course, initial orientation, ongoing training provided to mental health service staff; weekly group supervision meetings and individual as needed sessions with project manager.	Empathetic listening, share lived experiences, assist in recovery planning, link to community supports and services. Peers participated in mentoring support for each other and volunteers interested in becoming peer workers.	300 bed days saved, estimated cost savings A$93,150 during evaluation period. Participants emphasised importance of having someone who understands and provides positive role model. Referrers, carers, GPs and peers provided positive feedback about the program including, the warmth, understanding, credibility of peers, improvement in consumer care, communication between services, value of peer input in recovery-oriented practice. GPs highlighted the benefit of peers in helping them better understand patients’ symptoms and needs.	✓✓✓
Le Novere, 2023 [31]	Assess the cost effectiveness of program for crisis resolution team clients	NR	NR	NR	Authors concluded the intervention was cost effective and maintained over time.	NR
Leijdesdorff, 2022 [78]	Describe the @ease working method, present comprehensive profile of its visitors aged 12–25 during the organization’s first 2.5 years.	Young-adult peers, including experts by experience	2-day training (active listening skills, @ease’s working method, how to use own experiences, dealing with crisis, solution-focused and motivational conversation techniques) by peer workers. Supervision by healthcare professionals.	Young people are welcomed by a trained young adult peer. Experienced peer-workers involved in training the new young-adult peers.	NR	NR
Lucksted, 2013 [79]	Assess the benefits to family-to-family participants 6 months post completion of peer-taught family mental illness education program.	Family members of someone with a mental illness.	3-day training course on how to deliver curriculum as prescribed led by National Alliance for Mental Health state level trainers.	Training facilitation, provide a safe space where participants can learn to cope with their situation.	Benefits included: participants learning new skills, participants going on to support others or becoming class facilitators making the program self-sustaining, improved family relationships.	✓✓
Milton, 2017 [38]	Report on the development, feasibility of peer supported, self-management intervention for people leaving Crisis Resolution Teams services.	Peer with lived experience of mental illness	4-day course (meaning of peer support, listening, self-management and recovery, valuing diversity, story sharing, working with distress and addressing safety concerns), relevant NHS training; induction to the service. Group supervision by NHS Trust personnel.	Peers work with a participant in ten one to one sessions, facilitate the completion of a recovery plan, assist people to identify strategies to monitor warning signs, develop coping strategies, identify sources of help.	Participants were positive about peers’ intervention facilitation; they felt structured self-management booklet was underutilised but was a helpful framework, they raised concerns that clinical staff supervision of peers risked eroding their unique, non-clinical role, access to additional support from experienced peer support worker was advocated.	✓✓✓
Nasution, 2019 [80]	Determine the effects of cognitive behavioural therapy and peer leadership on suicidal ideation among adolescents in senior high school	NR	NR	Intervention facilitation	Although suicide ideation was reduced in both groups, in the first group (received peer support and CBT in additional to mental health nurses,) suicidal ideation decreased from high to none.	NR
Oostermeijer 2024 [81]	Report the impacts of residential short-term peer-support service for people 18–64, at risk of suicide, referred by a health professional.	NR	Open dialogue, befriending and trauma informed support training. Registered psychologist provided clinical oversight.	Peers and mental health support workers supported guests to make well-being, safety, and self-care plans. assist in completing assessments or contact relevant service	Peers were considered to be helpful, and a major contributor to feelings of connectedness, Peers increased the participants confidence to engage with other mental health services. Concerns mentioned about high staff rotation, possibility of traumatising staff.	✓✓
Ostrow 2015 [82]	Outline the implementation, research issues that peer respites face.	NR	Intentional Peer Support.	Outlines what a peer respite is, their mission and goals of fostering wellness, increasing meaningful recovery choices, creating and maintaining mutual and supportive relationships, reducing emergency hospitalizations and system costs.	Implementation complexities include ethical dilemmas, not admitting unstable housing guest who could benefit from their service, who would be discharged to the streets, the respite acting as a proxy homeless shelter; some respites exclude people in extreme states due to staffing, funding constraints limiting reach to help people. Careful recruiting of personnel that understand and work in a recovery model, guidelines that outline how they provide services to guests could help address the problem of fitting within a medial model. Research is needed to examine the processes, outcomes and costs of respites.	NR
Pelot, 2021 [83]	Describe the Peer Respite Essential Features survey data for 32 peer respite programs across 14 states in USA	People with lived experience of extreme mental health states.	The organisations reported training, Intentional Peer Support training (38%, n = 12) peer specialist training (50%, n = 16), and Wellness Recovery Action Planning (28%, n = 9).	Peer respites are run by peer staff, provide nonclinical community-based support for people experiencing or at risk of acute psychiatric crisis, operate 24 h per day, may be used as a diversion from emergency services or as a’ step-down from those settings, operate to build a community of people with shared experiences.	The flexibility peer respites offer could be essential in filling the gaps in the mental health system, need for future research to show their effectiveness in using less coercive support, to support the theory of crisis diversion of peer respites, compares service features, connects the fidelity of model to outcomes.	✓✓
Pfeiffer, 2019 [84]	Assess the acceptability, feasibility, fidelity of Peers for Valued Living intervention to reduce suicide risk for adults 18+ years, admitted to the inpatient psychiatry units	State certified peer workers, at least one-year professional peer specialist work experience, have personal lived experience of recovering from suicidal thoughts or a suicide attempt.	3-day program (peer support in the context of suicide, grief and loss, improving hope, managing acute suicide risk, empathy relaxation mindfulness, motivational interviewing techniques, strengthening support networks) through didactic sessions, group discussions, role-play exercises, and video demonstrations. Weekly group or ad hoc one to one supervision with clinician	Sharing lived experience, supportive listening, sharing recovery stories, improving hope, enhancing belongingness, managing acute suicide risk, using relaxation and mindfulness techniques, maintaining participant wellness, and motivational interviewing.	Participant feedback on peers was positive, they expressed satisfaction with the advice received, listening and support provided. Authors concluded that the findings suggest that the peer-led program was acceptable and feasible.	✓✓
Shattell, 2014 [85]	Describe the lived experience of guests and staff of community, recovery-oriented, alternative crisis intervention environment.	People with mental health experience.	Peers were trained	Peers provide hands-on support, talking about what guests hope to gain from being there, counselling about coping strategies, managing symptoms, case management, developing a plan to help them move forward.	Participants felt that the service was a quiet, safe environment in which they felt welcomed, could be themselves, could take their time, lack of clinical aspects meant they could relax, feel in control. Peers considered to be essential in creating a non-judgemental environment. Caring atmosphere was welcomed by peers with staff staying back after hours to discuss peers experience during shift. Misuse of the services was raised, staff mentioned guests coming when not in a crisis and guest saying they felt that the staff member did not agree with them about being in a crisis.	NR
Sheehan, 2023 [39]	Evaluate the peer-led strategic disclosure intervention for suicide attempt survivors, 18+ years, one-lifetime suicide attempt not within the past three months	Facilitators had lived experience of suicide and suicide ideation.	Six-hour, To Share or Not to Share? (2Share) strategic disclosure course, (disclosure pros and cons, ways to disclose, telling your story), two-day Honest, Open Proud (HOP) seminar; one-day facilitator training with certified HOP trainer; two practice sessions	Peers co facilitated the 2Share course.	Majority of participants attended all sessions; one participant left the intervention due to distress caused by the curriculum. Participants that subsequently had a disclosure experience reported positive experiences. Participants said being with people with similar experiences was important	✓✓✓
Smullen Thieling, 2022 [86]	Describe the peer-led program, the role of nurses in the Wellness Respite program for people, 18+ years, managing acute distress, implications of need for expanded services post COVID.	Majority of staff have lived experience of accessing behavioural health services.	Staff are trained to provide strengths-based wellness support	Peers support guests in exploring strengths in 14 wellness domains, serve as role models in maintaining balance through wellness and self-care strategies, help link to social networks, offer emotional, social, practical help, focus on valued life roles and individual capabilities, strengths.	Participants highlighted the value of a comfortable, safe environment in which to plan and set goals. Common problems were interactions between other guests and visitors that disturbed the respite’s atmosphere, some staff attitudes and the length of the intervention. Overall, participants were positive about the support provided by all the staff.	✓✓
Uren 2022 [87]	Provide an understanding of impact of peer-led mental health services through personal narrative	Relationships were based on connecting with other who have experienced similar struggles	NR	Peer support, experiential sharing, mentoring, advocacy, connection to resources, build a sense of community, coaching, develop meaningful relationships where person feels heard and understood.	Author stated that choice and increased availability of peer services are highly recommended as part of mental health service transformation. Mental health nurses can contribute to this success by advocating, valuing supporting peer involvement in mental health services.	✓
Wilson, 2022 [88]	Assess the feasibility, acceptability, preliminary effects of safety planning by peers in the Emergency Department	State certified peer worker with experience of suicidal ideation or survived a suicide attempt.	12 h training, biweekly feedback on the completeness and adherence to the intervention protocol, weekly debrief from clinical counsellor.	Safety planning.	Quality and completeness of plans differed significantly with the peer assisted plans being more complete and of higher quality than provider assisted plans. Participants equally liked making plans with peers and providers.	NR
Woodward 2023 [40]	Collaboratively develop and adapt safety planning intervention for peer-to-peer delivery in Arkansas rural communities, identifying implementation barriers and facilitators	No information on peer workers, working group members were veterans with or without prior experience with suicidal thoughts or attempts, support persons	Peers need intensive training, continued supervision and debriefing.	Safety planning intervention adaptation from health care providers in a clinical setting delivery to peers in community settings delivery.	No significant changes needed to intervention. Recommendation for robust training for the peers which included suggestions on the content of the training. Participants outlined 27 facilitators and 47 barriers to implementing peer interventions. Facilitators included peers’ acceptability, timely nature of assistance, organisations established network and ability to train peers. Barriers included not enough peers, availability and regularity of training, uncertainty in reaching veterans in need.	✓✓✓
Wusinich, 2020 [89]	Describe the impact of Parachute program for people 16+ years with a diagnosis of a serious mental illness, on enrolees, support persons and network members.	NR	Parachute team members received training in Open Dialogue and Intentional Peer Support (IPS).	The team including peer specialists used open dialogue to discuss the participant and their network’s current situation, challenges, and concerns	Most of the participants were satisfied with the service and members of the team.	✓✓

Key: ✓ Peer or co-design involvement in conduct of research only, ✓✓ Peer or co-design involvement in intervention or program development, ✓✓✓ Peer or co-design involvement in both intervention development and conduct of the research, NR = Not reported/unclear from the paper.

### 3.2. Peer Worker Description

Descriptions of peer workers in the articles ranged from detailed (e.g., including age range, whether they were certified peer workers or had previous peer work experience) to minimal (noting only lived experience of mental health or history of suicide attempts or ideation). A small number [31,44,52,69,73,80,81,82,89] did not report peer worker characteristics, primarily in the suicide related articles. Specificity of the information was also similar in both groups although descriptions were often vague: for example reporting that the peer workers *“had experience working at an inpatient psychiatric clinic”* (p. 4) [56]; that peer workers were prisoners involved in a prison therapeutic community [74]; and mentioning a trained facilitator with no description of whether lived experience was required [71]. 

### 3.3. Training and Supervision

Thirteen articles (three non-suicide-specific [52,62,63], ten suicide related [31,32,34,67,68,69,75,77,80,87]) provided no information on either training or supervision. One non-suicide-specific distress article specifically stated the peers were not trained and gave no information on supervision [53], and another stated that apart from training on child protection procedures and confidentiality, no structured training program was followed because the peer workers *“strongly felt that they wanted to provide the intervention simply as a fellow mum”* (p. 39) [48].

The level of information given on training was limited in both groups. For example, only 23 articles (12 non-suicide-specific [42,43,44,45,50,51,54,55,58,59,60,61], 11 suicide related [33,37,38,39,64,65,66,78,79,84,88]) specified the duration of training, and only 3 described the conduct of training in detail [50,61,84]. 

Similar proportions of both groups of articles (7 non-suicide-specific [43,47,50,54,56,57,59], 12 suicide related [35,36,37,38,39,70,78,81,82,83,84,89]) included training on peer-specific topics and skills. The three articles that included peer-specific topics and skills were delivered by clinical professionals [43,44,61] were all non-suicide specific articles, and the two articles explicitly stating that the training was conducted by peers [65,78] were suicide related. Several articles mentioned training on motivational interviewing, which is not consistent with the non-clinical orientation and mutuality of peer work. This was included in the training in more of the non-suicide specific articles [42,50,58] than the suicide related articles [84].

Only three articles specifically mentioned supervision by peers [33,35,51], and another mentioned supervision by a clinician and a peer [41]. Similar numbers of articles (five non-suicide-specific [43,44,50,54,56], six suicide related [33,76,78,81,84,88]) explicitly stated that supervision was conducted by a clinician. The other articles included supervision provided by supervisors [55,57], researchers [49], project managers [37], intervention trainers [42,64] or did not mention who conducted the supervision [65].

### 3.4. Components of the Peer Model 

Two suicide related distress articles [31,34] did not supply any information on the peer model, and one non suicide specific article mentioned that peers were asked their opinion on the model, but no information was given on the model itself [62]. Seventeen articles (ten non-suicide-specific [42,43,44,45,46,47,55,59,61,63], seven suicide related [39,64,65,73,74,80,88]) only mentioned that peers facilitated the intervention. 

In general, more of the suicide related articles provided specifics on model components, especially providing support [35,36,49,56,59,64,67,69,70,72,75,77,84,85,86,87], safety and recovery planning [33,35,37,38,48,50,52,67,68,76,77,81,85,88], advocacy [68,87] and developing skills [32,36,38,43,44,49,55,64,67,69,75,76,84,86]. Only four articles described creating a safe and supportive environment (three suicide related [68,79,87], one non-suicide-specific [47]). A similar number in both groups said that they provided information as a component of their peer model (five non-suicide-specific [41,43,44,50,59], six suicide related [38,66,69,71,75,77]).

### 3.5. Outcomes

The primary outcomes reported were measures of satisfaction for recovery, practical and/or emotional support, provision of information and social connection/interaction. More suicide related articles reported satisfaction with recovery outcomes (five non-suicide-specific [42,47,52,56,58], nine suicide related [32,35,67,68,71,75,77,80,88]) and satisfaction with practical and/or emotional support (two non-suicide-specific [53,60], five suicide related [35,66,77,80,84]). A similar number of articles in both groups mentioned satisfaction with the provision of information [34,43,58,60,66] and social connection/interaction [52,67,71]. Cost effectiveness was only measured in four suicide related articles [12,31,35,37], and two suicide related articles specifically mentioned participants’ satisfaction with the safe environment provided [85,86].

Reporting of positive feedback on peer support was similar in both groups. However, the suicide related articles also included information on the constraints and concerns. These included staff retention and trauma [81], the professionalisation and commodification of peer work and the subsequent need to work within a broken system [70] and a lack of engagement and resistance from some consumers and families [65]. One non-suicide-specific distress article had mixed feedback, noting that negative peer attitudes negatively affected recovery attitudes [53]. 

Two articles studied peer self-disclosure, one around disclosure focussed on suicide [36] describing how peers conveyed lived experiences related to suicide while maintaining a message of recovery, and the other focussed on distress and depression [61], where peers shared personal experiences to guide client action. 

## 4. Discussion

The scoping review sought to identify the design, characteristics and evidence for peer-led models and interventions for people experiencing emotional distress and/or suicidal crisis. The review provides a comprehensive overview of interventions that focus on emotional distress or crisis in addition to suicidality and specifically focusses on services that are led, informed, co-designed, or co-facilitated by peers with lived experience of emotional distress and/or suicidal crisis. The information provided on peer models was varied, with a third of the articles having no information or only stating that a peer worker facilitated the intervention, which means findings should be interpreted with caution. Most studies that did include the relevant information, regardless of their specific setting, target population or design, suggested that peer-led programs were well accepted and achieved positive outcomes, predominantly in the form of improvements to mood, social connectedness, communication and coping skills. The provision of emotional and crisis support, recovery planning and developing skills were the main facilitators of positive outcomes. 

A key element of effective peer work identified across numerous systematic reviews is the importance of role modelling via shared identity [22]. Recent research also indicates that peer workers within non-clinical suicide prevention services require specialised crisis skills and experience [9]. Several studies in both groups of our review (e.g., [37,39,54,56,65]) reported that positive outcomes were associated with shared experience, promoting a sense of understanding and credibility for peer work roles. There is evidence to suggest that peer work may be more effective in certain populations, such as perinatal mothers [22]. The relative lack of information on the peers involved in the models was therefore surprising: less than half of the studies included detailed descriptions, and many used vague descriptors that did not clearly indicate if the peer workers involved had closely related experiences to service users. Whether this is a reporting issue, such as assuming the characterisation of a peer is self-explanatory, or a reflection of a more significant problem with models implementing appropriate peer roles, is not clear. However, this lack of precision around peers within current literature limits our ability to draw accurate conclusions about their effectiveness and undermines the advancement of the field of peer work. Thus, the failure to include details on this key element of effective peer work is a substantial gap in the research evidence for distress-related peer model research.

The availability of peer skills training and supervision is key for effective peer work and the long-term stability of peer programs [25,32,34,40]. Training and supervision were mentioned in approximately 78% of the surveyed articles. Whilst there was some information provided on the peer-specific skills training, the content of this training was either absent or not specific to the peer work role. Training was typically related instead to the delivery of the intervention and/or training that was broad-based (cultural awareness, active listening, strengths-based wellness support). This emphasis on professional training in intervention delivery is more reflective of a process of professional oversight, as opposed to supportive supervision in peer work. In addition, in some cases, including programs where training in specific peer work skills was evident, there was a discordance between training and peer work principles, such as training in motivational interviewing or counselling skills [90]. This conflation may position peers as a type of informal clinician, particularly undermining core peer work principles such as mutuality and reciprocity. In addition, there were also interventions where training was delivered by non-peer workers, including psychiatrists, clinical psychologists and nurse practitioners, further undermining the specialism of peer work. 

Results from this study attest to the challenges peer workers face in navigating the complex and sometimes competing sets of negotiations and accommodations regarding the expectations and needs of consumers and health professionals [91]. A key strength of peer approaches is the ability to work flexibly and in nontraditional ways, to forge trusting relationships with consumers, especially those who have been marginalised or harmed by services, and to be open to the views of others on difficult issues such as the use of medication and the desire to die [9,92]. Moreover, the exercising of stringent organisational care mandates within health systems and knowledge fostered by dominant professional groups can dilute the authenticity of peer work and compromise practice, reinforcing the power of clinical knowledge and maintaining the status quo [93,94]. Training that is not reflective of peer work practice has been identified as a barrier to the implementation of peer models and may lead to role ambiguity and role conflict, impacting peer worker wellbeing [25,95]. Role concerns were also reflected in some peer workers’ views about the suitability and effect of clinical supervision on their professional development and whether additional supervision from an experienced peer worker was required [38].

A significant limitation in the reviewed literature was the unspecified role that peers, consumers and carers had in contributing to the design of services/interventions or the research itself. Over half of the articles made no mention of peer, consumer or carer involvement. Whether this omission was an error in reporting, reflecting poor transparency, or indicative of a lack of engagement is unclear. Co-design, the collaborative process by which diverse stakeholders, including consumers, carers and health professionals, actively participate in research, service, program and policy design and delivery, has become a hallmark of best practice in mental health care [96,97]. The lack of inclusion of lived experience in co-design, particularly in suicide prevention, raises significant ethical concerns in peer model development, including risks of a lack of suitability and appropriateness of programs, and ensuring the prevention of unintended harms. In addition, because many of the studies focused on facilitation of the service model or intervention rather than on the peer model itself, there was limited information on underlying rationales for action and the empirical and ethical arguments that supported these rationales. 

Additional gaps in knowledge centre on the importance of health care-built environments for people experiencing emotional distress and/or suicidal crisis. Consumer-led and focused research has consistently reported on the harms that take shape in clinical settings and crosscutting issues of safety and quality of care [98]. The creation of safe, comfortable, and supportive environments needs to be a primary focus of service models and interventions. However, for the most part, the effects of the built environment on mental health, well-being, and recovery were left underexplored in these articles. 

### Limitations

The limitations include that only articles in English were reviewed, and most (43/59) of the articles included in the analysis were from the USA and the UK. Issues specific to location likely affected the design and implementation of the peer models; thus, the applicability of the findings to other countries or health care systems may be limited.

## 5. Conclusions

The evidence base for peer models of care for emotional distress and suicidal crisis is still developing, making conclusions preliminary. However, this review broadly found that peer-led programs were well accepted, and evidence suggested they achieved positive outcomes, including improvements to mood, social connectedness, communication and coping skills. Non-clinical, peer-based interventions are a rapidly growing component of mental health care and suicide prevention. Their emergence coincides with the international expansion of peer work within community mental health and growing recognition of the importance of alternative spaces for mental health care [13,14]. Empirical gaps with current evidence remain, including a focus on reporting the facilitation of the service model or interventions and not on the peer models themselves. In addition, practice gaps currently include a lack of the provision of peer-specific training, appropriate supervison and explicit use of co-design; although, the lack of these may also reflect inadequate reporting. This limits both empirical and ethical arguments on the use of these models in practice. Future research into peer-led models should focus on addressing these empirical and practice gaps, using genuine co-production approaches to develop standards for implementation, evaluation and reporting that are grounded in the principles of respect, mutuality and non-clinical orientation. 

## Figures and Tables

**Table 1:** Inclusion and exclusion criteria.

Inclusion	Exclusion
Original criteria	
People above the age of 16 years who are experiencing emotional distress and/or suicidal crisis—including people with a mental health diagnosis. Provision of emotional/psychosocial support, or crisis intervention services with primary outcomes related to: distress, connection/safety/empowerment, suicide prevention, hospital diversion/avoidance or service experience. Services that involve interaction between users and facilitators (including asynchronous interactions) Explicitly mentions that services are led, informed, co-designed or co-facilitated by peers with lived experience of emotional distress and/or suicidal crisis. (‘peers’ includes individuals with personal experience and/or those who have supported a family member, friend, or close other experiencing distress). Published in the English language	Articles that are purely self-directed/self-help resources. Articles relating to purely gate-keeper programs or similar e.g., mental health literacy, mental health first aid, suicide awareness training programs. Articles that outline informal peer supports that lack defined principles/processes/organised models/structure/program. Articles concerning a primary outcome of psychiatric symptoms only. Media reports. Books.
Second round criteria ^1^	
	Articles that are not explicitly about distress. Articles where peers are not explicitly health/disability related peers. Documents that are not peer reviewed articles

^1^ The second round criteria represented a narrower application of the criteria. These were used in a second title and abstract screening in the initial search and in the titles and abstract screening for the updated search. See Screening and Identification of Studies below for further information.

## Data Availability

The data analyzed in this review are included in the article/supplementary material. Further inquiries can be directed to the corresponding author.

## Supplementary Materials

The following supporting information can be downloaded at: https://www.mdpi.com/article/10.3390/ijerph23020273/s1. Supplementary Table S1 PRISMA-ScR (extension for Scoping Reviews) reporting checklist. Supplementary Table S2: Key search terms and search strategy. Supplementary Table S3: Characteristics of peer models of support for people experiencing suicidal crisis, ideation and distress articles (n = 37). Supplementary Table S4: Characteristics of emotional distress without explicit mention of suicidal ideation and crisis articles (n = 22). Reference [99] is cited in the supplementary materials.

## Abbreviations

The following abbreviations are used in this manuscript:

MeSH	Medical Subject Headings
PRISMA	The Preferred Reporting Items for Systematic reviews and Meta-Analyses extension for Scoping Reviews
PCC	Participants, Concept, Context
PTSD	Post traumatic stress disorder
